# Successful treatment discontinuation in CML patients with full-dose and low-dose TKI: Results from real-world practice

**DOI:** 10.3389/fphar.2023.1101743

**Published:** 2023-01-23

**Authors:** Yilin Chen, Huifang Zhao, Jingming Guo, Jing Zou, Wenjuan He, Danlei Han, Fanjun Cheng, Yanli Zhang, Weiming Li

**Affiliations:** ^1^ Department of Hematology, Union Hospital, Tongji Medical College, Huazhong University of Science and Technology, Wuhan, Hubei, China; ^2^ Department of Hematology, The Affiliated Cancer Hospital of Zhengzhou University, Zhengzhou, Henan, China; ^3^ Department of Hematology, Yichang Central People’s Hospital & First Clinical Medical College of China Three Gorges University, Yichang, Hubei, China

**Keywords:** chronic myeloid leukemia, tyrosine kinase inhibitor, dose reduction, treatment free remission, real-world practice

## Abstract

**Background:** In clinical studies, some patients who achieve deep molecular response (DMR) can successfully discontinue tyrosine kinase inhibitor (TKI). TKI dose reduction is also an important aspect of alleviating adverse effects and improving quality of life. This study aimed to explore the outcome after drug withdrawal in Chinese CML patients.

**Methods:** We conducted a retrospective analysis of the outcome of 190 patients who stopped TKI. 27 patients experienced dose reduction before TKI discontinuation. The median duration of TKI treatment and MR4 before discontinuation was 82 months and 61 months.

**Results:** With median follow-up after stopping TKI treatment of 17 months, the estimated TFR (Treatment Free Remission) were 76.9% (95%CI, 70.2%–82.4%), 68.8% (95%CI, 61.3%–75.2%), and 65.5% (95%CI, 57.4%–72.5%) at 6, 12 and 24 months. For full-dose and low-dose TKI groups, the TFR at 24 months was 66.7% and 55.8% (*p* = 0.320, log-rank). Most patients (56/57) quickly achieved MMR after restarting TKI treatment. Multivariable analysis showed that patients with TKI resistance had a higher risk of molecular relapse than patients without TKI resistance (*p* < 0.001).

**Conclusion:** TFR rates were not impaired in patients experiencing dose reduction before TKI discontinuation compared to patients with full-dose TKI. Our data on Chinese population may provide a basis for the safety and feasibility of TKI discontinuation, including discontinuation after dose reduction, in clinical practice.

## 1 Introduction

The advent of tyrosine kinase inhibitor (TKI) has revolutionized the treatment of chronic myeloid leukemia (CML) and improved the prognosis of patients to near-normal levels (Sasaki et al., 2015). However, the adverse effects associated with long-term TKI therapy are an overwhelming factor affecting the quality of life (Steegmann et al., 2016). Besides, the high cost of persistent TKI treatment is increasingly becoming an important issue (Phuar et al., 2019). Over recent years, numerous clinical studies have proved that about 40%–60% of patients with deep molecular response (DMR) could achieve TFR (Rea et al., 2017b; Etienne et al., 2017; Mahon et al., 2018; Saussele et al., 2018; Nagafuji et al., 2019; Kimura et al., 2020; Shah et al., 2020). In this context, treatment-free remission (TFR) is a major goal for all CML patients according to the current guidelines (Hochhaus et al., 2020). Recently, The Gruppo Italiano Malattie EMatologiche dell’Adulto (GIMEMA; Italian Group for Hematologic Diseases of the Adult) CML Working Party developed a project designed to select treatment policies that might increase the likelihood of achieving TFR. A consensus was reached on disease risk assessment, first-line treatment, and the responses that require a change of the TKI, contributing to optimizing the treatment strategy for TFR (Baccarani et al., 2019). However, data on the impact and outcomes of TKI discontinuation outside of clinical studies remain sparse. In clinical practice, TKI dose reduction is also an important aspect of alleviating adverse effects and improving quality of life (Russo et al., 2015; Iurlo et al., 2020). In addition, several studies have shown that low-dose TKI treatment is effective in maintaining major molecular response (MMR) (Rea et al., 2017a; Clark et al., 2017; Claudiani et al., 2021). Moreover, dose-halving therapy yields higher molecular recurrence-free survival rate compared to direct TKI discontinuation (Luo et al., 2022). Our recent study also confirmed the feasibility of TKI dose reduction therapy for Chinese CML patients (Chen et al., 2022). The concern of whether dose reduction before TKI discontinuation affects patient’s access to TFR is also of interest to investigators. The DESTINY study has investigated the effects of low-dose TKI treatment withdrawal before stopping TKI, of which patients with MMR at dose reduction experienced a TFR rate of 36% at 36 months and patients with DMR reported a TFR rate of 72% (Clark et al., 2019), indicating TKI de-escalation is safe for most patients with DMR. TKI de-escalation - to discontinue TKI may be an alternative way to achieve TFR (Kunbaz and Eskazan, 2019). Still, more studies are needed to investigate the possibility of TKI discontinuation in patients with dose reduction, prior treatment failure, or other problems in the real world. In this study, we retrospectively analyzed a real-world series of patients with CML who discontinued TKI, including patients who received low-dose TKI therapy prior to discontinuation.

## 2 Materials and methods

### 2.1 Patients

We collected data from CML-CP patients who discontinued TKI between June 2013 and July 2022 from the three hospitals in China (Union Hospital, Tongji Medical College, Huazhong University of Science and Technology; The Affiliated Cancer Hospital of Zhengzhou University; Yichang Central People’s Hospital & First Clinical Medical College of China Three Gorges University). All patients were diagnosed with CML-CP by bone marrow cell morphology, cytogenetics, and molecular biology examination. All patients who discontinued TKI were treated with TKI for at least 3 years, and MR4 lasted at least 2 years.

### 2.2 Molecular response definitions

Molecular monitoring was assessed by quantitative polymerase chain reaction (qPCR). The *BCR-ABL 1* level was expressed on the international scale (IS). The Molecular response was assessed according to ELN criteria (Hochhaus et al., 2020). In detail, MMR was defined as a *BCR-ABL/ABL* ratio ≤0.1%, and MR4 was defined as a *BCR-ABL/ABL* ratio ≤0.01%. Molecular relapse was defined as loss of MMR. TFR was calculated from TKI discontinuation to the loss of MMR.

### 2.3 Statistical analysis

Comparison of continuous variables was tested by Student t-test or Mann-Whitney *U* test, and comparison of categorical variables was tested by χ^2^ or Fisher exact test. TFR was estimated using the Kaplan-Meier method, and comparison between groups was used log-rank tests. Univariate analysis of potential clinical factors associated with molecular relapse was performed using the Kaplan-Meier method. Multivariate analysis was performed using the Cox regression analysis. Proportionality hazards assumptions were checked for factors included in Cox regression analysis. All analyses were carried out using SPSS, version 22 statistical software, and GraphPad Prism 7.

## 3 Result

### 3.1 Patient characteristics

**FIGURE 1:**
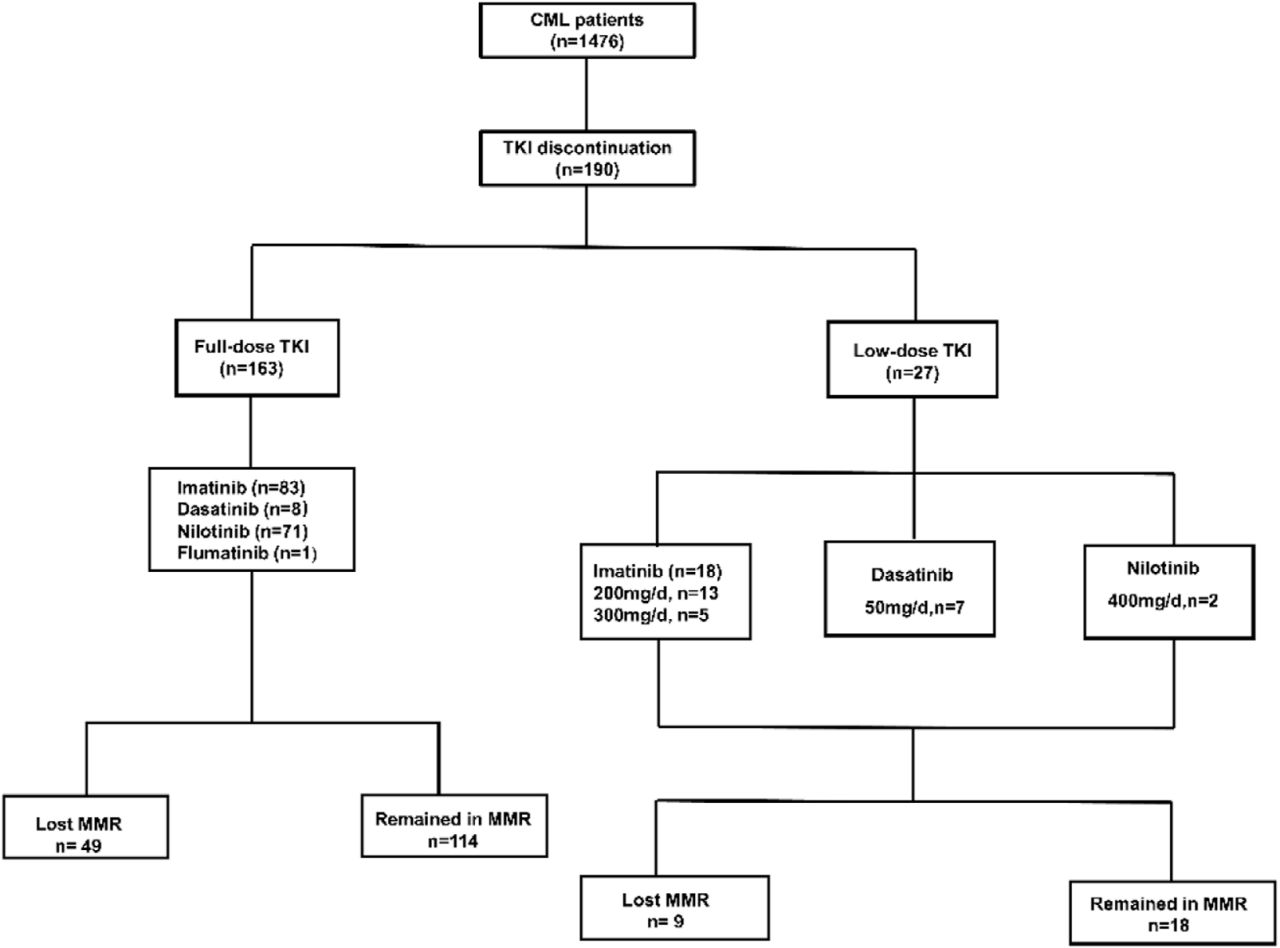
Study population flowchart. TKI, tyrosine kinase inhibitor.

Among 1476 CML patients, 190 patients who discontinued TKI were included in this study, 97 of whom were female and 93 were male, the median age was 43 years (range, 3–74 years) (Figure 1, Table 1). 182 patients were on imatinib at diagnosis, of which 81 patients switched to second-generation TKI for resistant or non-resistant reasons. Eight patients were on a second-generation TKI (2G-TKI) at diagnosis. 101 patients took imatinib, 15 patients took dasatinib, 73 patients took nilotinib and 1 patient took Flumatinib at discontinuation, respectively. The median duration of TKI treatment before discontinuation was 82 months (range, 36–222), the duration of MR4 before TKI discontinuation was 61 months (range, 24–162), and the time to MR4 was 12 months (range, 3–112). Twenty seven patients underwent dose reductions before TKI discontinuation. Forty eight patients experienced TKI resistance (imatinib, *n* = 48) before TKI discontinuation according to ELN criteria (Hochhaus et al., 2020).

**TABLE 1:** Patient characteristics.

	Full-dose (*n* = 163)	Low-dose (*n* = 27)	Overall (N = 190)	*p*
Median age (range), years	43 (3–74)	49 (5–65)	43 (3–74)	0.387
Male, n (%)	83 (50.9)	14 (51.9)	97 (51.1)	0.929
TKI at TKI discontinuation (n%)				
imatinib	83 (50.9)	18 (66.7)	101 (53.2)	<0.001
dasatinib	8 (4.9)	7 (25.9)	15 (7.9)	
nilotinib	71 (43.6)	2 (7.4)	73 (38.4)	
flumatinib	1 (0.6)	1 (0.5)		
Current TKI therapy line, n (%)				
1st	90 (55.2)	18 (66.7)	109 (57.4)	0.437
2nd	69 (42.3)	9 (33.3)	77 (40.5)	
3rd	4 (2.5)	x	4 (2.1)	
Sokal score, n (%)				0.053
Low	47 (28.8)	5 (18.5)	52 (27.4)	
Intermediate	24 (14.7)	5 (18.5)	29 (15.3)	
High	11 (6.7)	6 (22.2)	17 (8.9)	
NA	81 (49.7)	11 (40.7)	92 (48.4)	
ELTS score, n (%)				0.484
Low	52 (31.9)	9 (33.3)	61 (32.1)	
Intermediate	23 (14.1)	4 (14.8)	27 (14.2)	
High	7 (4.3)	3 (11.1)	10 (5.3)	
NA	81 (49.7)	11 (40.7)	92 (48.4)	
History of TKI resistance, n (%)	40 (24.5)	8 (29.6)	48 (25.3)	0.573
IFN-α before TKI, n (%)	11 (6.7)	3 (11.1)	14 (7.4)	0.422
TKI duration before discontinuation: (range), months	82 (36–222)	83 (43–162)	82 (36–222)	0.887
Time to MR4 (range), months	12 (3–112)	17 (3–90)	12 (3–112)	0.606
MR4 duration before TKI discontinuation (range), months	62 (24–162)	58 (24–159)	61 (24–162)	0.427
Dose reduction n, %			27 (14.2)	

TKI, tyrosine kinase inhibitor.

### 3.2 Low-dose TKI treatment

In clinical practice, TKI dose reduction is also an important aspect of alleviating adverse effects and improving quality of life. Twenty seven patients underwent dose reductions before TKI discontinuation for adverse events (*n* = 17), desire to stop TKI (*n* = 8), and finical burden (*n* = 2) (Table 2). The median duration of low-dose TKI treatment was 18 months (range, 3–72). Overall, 22 patients experienced half of the standard-dose treatment. All 27 patients were MR4 or deeper at the time of TKI discontinuation. The median duration of TKI treatment before discontinuation was 83 months (range, 43–162), duration of MR4 before TKI discontinuation was 58 months (range, 24–159), and the time to MR4 was 17 months (range, 3–90) (Table 2).

**TABLE 2:** Reasons and dose for patients with dose reduction.

Patients no.	MR at time of dose reduction	Reason for dose reduction	Low-dose TKI	Duration of low-dose TKI (month)
1	MR4	leukopenia	Imatinib,300 mg/d	55
2	Not in MMR	leukopenia	Imatinib,300 mg/d	66
3	MR4	edema	Imatinib,200 mg/d	15
4	MR4	edema	Imatinib,200 mg/d	12
5	MR4	finical burden	Imatinib,200 mg/d	33
6	MR4	anorexia	Imatinib,200 mg/d	11
7	MR4	anemia	Imatinib,200 mg/d	12
8	MR4	anemia, fatigue	Imatinib,300 mg/d	39
9	MR4	desire to stop TKI	Imatinib,200 mg/d	23
10	MR4	desire to stop TKI	Imatinib,200 mg/d	3
11	MR4	anemia	Imatinib,200 mg/d	23
12	MR4	edema	Imatinib,200 mg/d	7
13	MR4	elevated bilirubin	Nilotinib, 400 mg/d	24
14	MR4	desire to stop TKI	Dasatinib, 50 mg/d	19
15	MR4	pleural effusion	Dasatinib, 50 mg/d	20
16	MR4	desire to stop TKI	Dasatinib, 50 mg/d	4
17	MR4	desire to stop TKI	Imatinib,200 mg/d	38
18	MR4	pericardial effusion	Nilotinib, 400 mg/d	3
19	MR4	fatigue	Imatinib,200 mg/d	18
20	MR4	desire to stop TKI	Imatinib,200 mg/d	24
21	MR4	pleural effusion	Dasatinib, 50 mg/d	14
22	MR4	pleural effusion	Dasatinib, 50 mg/d	21
23	MR4	finical burden	Dasatinib, 50 mg/d	20
24	MR4	desire to stop TKI	Dasatinib, 50 mg/d	12
25	MR4	desire to stop TKI	Imatinib, 300 mg/d	72
26	MR4	edema	Imatinib, 300 mg/d	3
27	MR4	fatigue	Imatinib, 200 mg/d	17

MR, Molecular response.

### 3.3 Discontinuation in patients with full-dose and low-dose TKI

**FIGURE 2:**
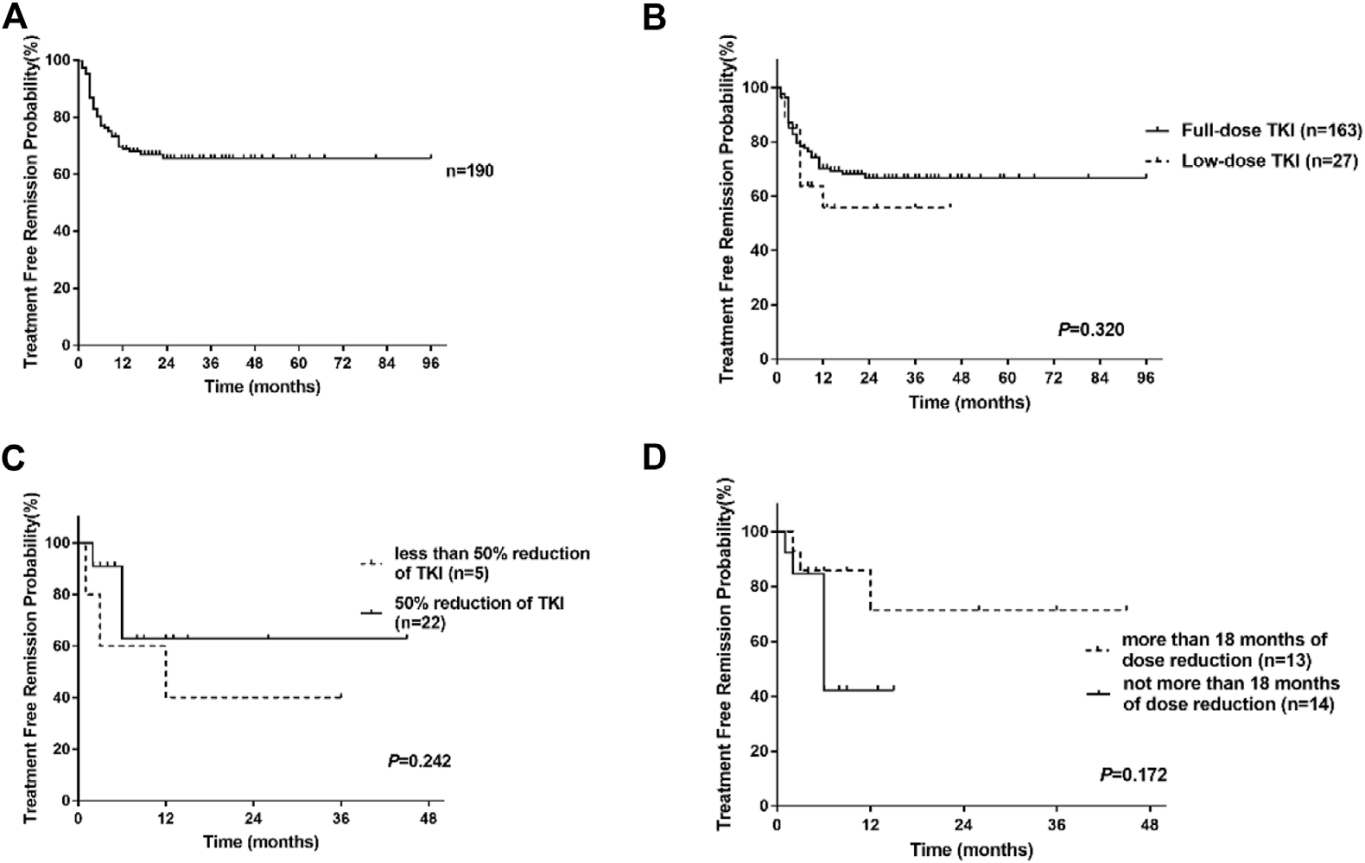
Treatment-free remission (TFR) after tyrosine kinase inhibitor (TKI) therapy. **(A)** TFR in the overall patients. **(B)** TFR in patients according to the dose of TKI. **(C)** TFR in patients with low-dose TKI according to the degree of TKI reduction. **(D)** TFR in patients with low-dose TKI according to the duration of dose reduction.

With median follow-up after stopping TKI treatment of 17 months (range, 2–96 months), 58 patients lost MMR, nine patients lost MR4, and 123 patients remained MR4 or deeper. The estimated overall TFR were 76.9% (95%CI, 70.2%–82.4%), 68.8% (95%CI, 61.3%–75.2%), and 65.5% (95%CI, 57.4%–72.5%) at 6, 12 and 24 months (Figure 2A). For full-dose and low-dose TKI groups, the TFR at 6 months were 78.4% (95%CI, 71.3%–84.0%) and 63.8% (95%CI, 39.6%–80.4%), the TFR at 12 months were 70.3% (95%CI, 62.3%–76.9%) and 55.8% (95% CI, 30.3%–75.2%), the TFR at 24 months were 66.7% (95%CI, 58.0%- 74.0%) and 55.8% (95% CI, 30.3%–75.2%) (Figure 2B). Hence, patients with low-dose TKI before TKI discontinuation achieved TFR rates similar to those of patients treated with maintenance full-dose TKI. Moreover, 81.5% (22/27) of low-dose patients experienced half of the standard-dose treatment. Those patients experienced a similar TFR rate compared to patients with less than 50% reduction in TKI (*p* = 0.242, log-rank) (Figure 2C). Moreover, we found that patients with at least 18 months of dose reduction achieved a similar TFR rate to those with less than 18 months of dose reduction (*p* = 0.172, log-rank) (Figure 2D). Our data showed that TKI de-escalation did not impact TFR.

### 3.4 Predictive factors for molecular relapse

**FIGURE 3:**
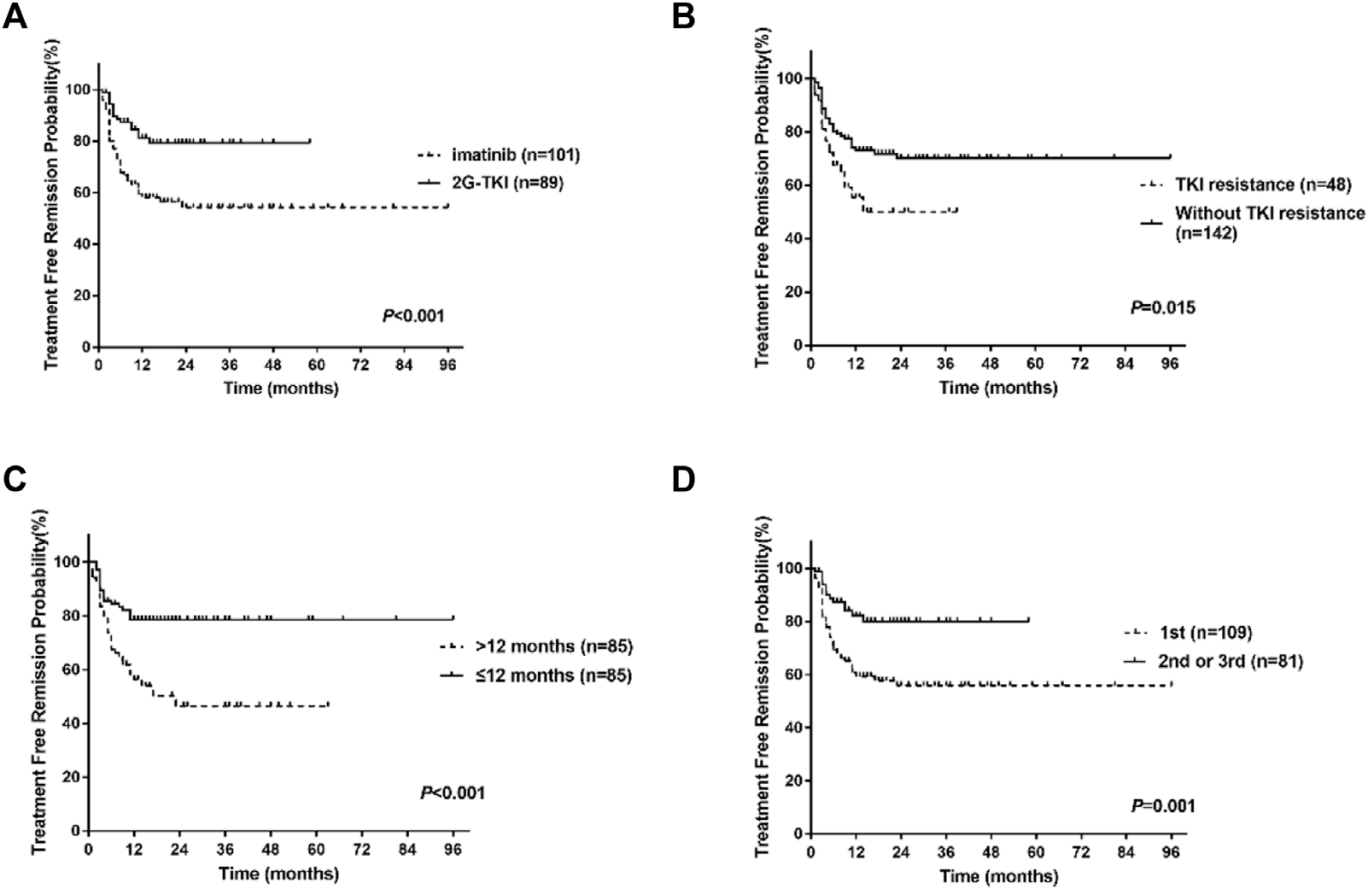
Treatment-free remission (TFR) after tyrosine kinase inhibitor (TKI) therapy. **(A)** TFR in patients according to the type of TKI at discontinuation. **(B)** TFR in patients according to TKI resistance. **(C)** TFR in patients according to the time to MR4. **(D)** TFR in patients according to the line of TKI. 2G-TKI, second-generation TKI.

**FIGURE 4:**
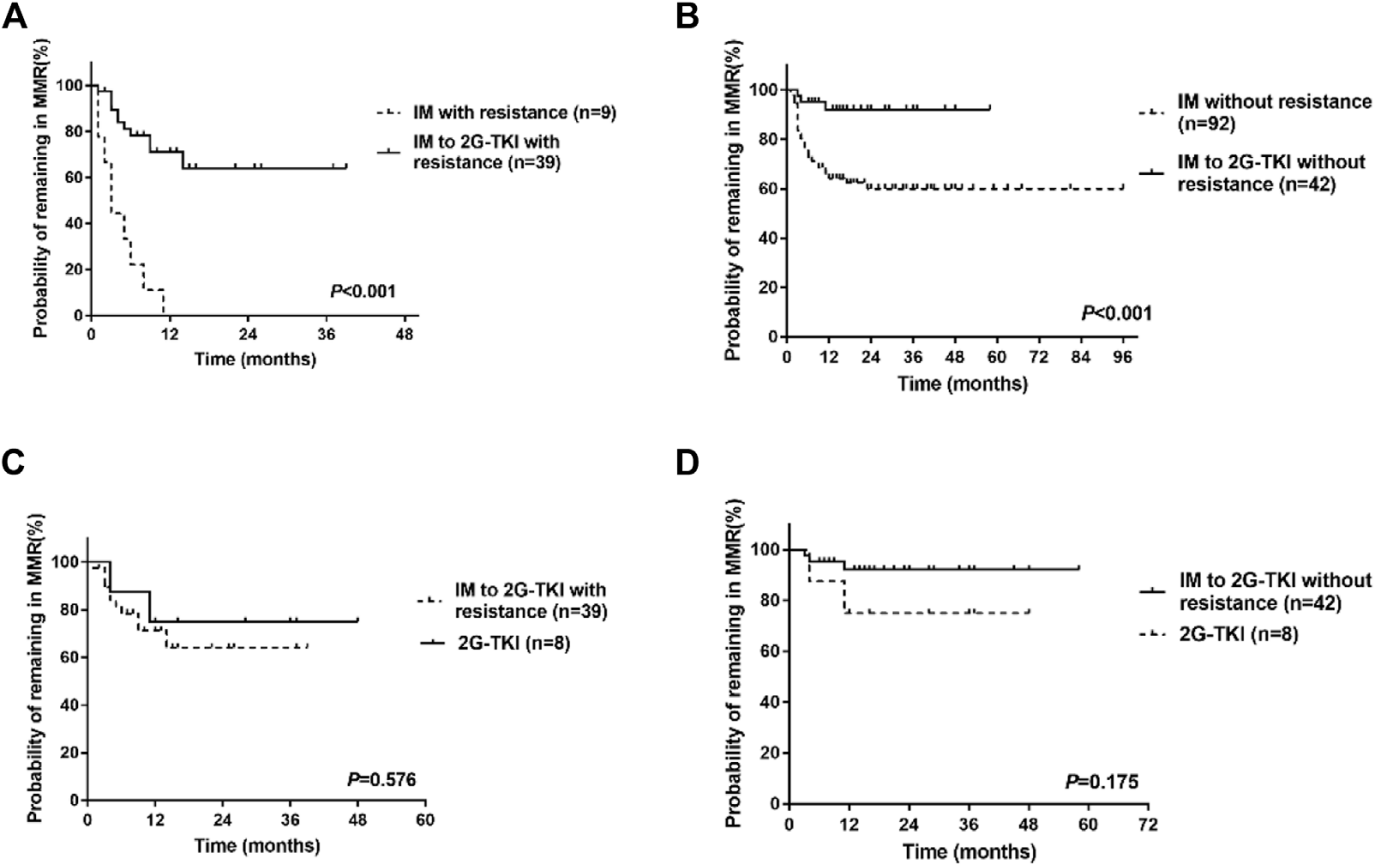
Treatment-free remission (TFR) in patients after tyrosine kinase inhibitor (TKI) therapy. **(A)** TFR in patients with imatinib resistance. **(B)** TFR in patients without resistance. **(C)** TFR in patients who switched to 2G-TKI for imatinib resistance or patients with 2G-TKI. **(D)** TFR in patients who switched to 2G-TKI therapy without imatinib resistance or patients with 2G-TKI. IM, imatinib; 2G-TKI = second-generation TKI.

Univariate analysis showed that the type of TKI at discontinuation, TKI resistance, line of therapy, and time to MR4 was the clinical variable significantly associated with molecular recurrence (Table 3; Figure 3). Multivariate analysis showed that only a history of TKI resistance was significantly associated with molecular recurrence. We performed a subgroup analysis to compare the differences in TFR among patients with different TKI treatments. We divided the patients into five groups: patients with imatinib-resistant who switched to 2G-TKI therapy (*n* = 39) or not (*n* = 9), patients without imatinib-resistant who switched to 2G-TKI therapy (*n* = 42) or not (*n* = 92) and patients treated with 2G-TKI in the first line (*n* = 8). For patients with imatinib resistance, those who switched to 2G-TKI therapy had remarkably higher TFR rates than those who did not (*p* < 0.001, log-rank) (Figure 4A). Similarly, patients without resistant who switched to 2G-TKI experienced higher TFR rates than those on imatinib treatment (*p* < 0.001, log-rank) (Figure 4B). We also found patients who switched to 2G-TKI therapy for imatinib resistance or non-resistance reasons achieved similar TFR rates compared to patients treated with 2G-TKI in the first line (Figures 4C, D). These results indicated that 2G-TKI therapy may yield higher TFR rates than imatinib in both imatinib-resistant and non-imatinib-resistant patients. In addition, TKI discontinuation should be attempted with great prudence in imatinib-resistant patients who maintaining imatinib therapy.

**TABLE 3:** Univariate and multivariable analysis of various parameters’ associations with molecular relapse.

Characteristic	Probability of remaining MMR at 2 years	*p* (Log-rank)	Multivariate analysis HR (95% CI)	*p*
Age, years		0.442		
≤43	68.1 (55.7–77.7)			
>43	62.9 (51.2–72.5)			
Sex (		0.056		
Male	58.5 (46.4–68.7)			
female	73.0 (61.9–81.3)			
Previous IFN-α treatment		0.578		
Yes	52.4 (16.2–79.5)			
No	66.8 (58.6–73.7)			
TKI at discontinuation		0.001		
Imatinib (ref.)	54.3 (43.0–64.3)		0.585 (0.140–2.436)	0.461
2-G TKI	79.3 (68.1–86.9)			
TKI resistance		0.015		
Yes	50.0 (31.8–65.7)			
No (ref.)	70.2 (61.0–77.6)		3.892 (1.811–8.365)	<0.001
Line of therapy		0.003		
1st (ref.)	56.5 (45.7–66.0)		0.298 (0.062–1.443)	0.133
≥2nd	78.8 (66.8–86.8)			
Sokal		0.791		
Low	72.5 (58.0–82.8)			
Intermediate	60.8 (33.8–79.6)			
High	56.0 (24.9–78.5)			
ELTS		0.672		
Low	73.3 (60.1–82.7)			
Intermediate	54.6 (30.0–73.7)			
High	67.5 (29.1–88.2)			
Duration of TKI, mo		0.736		
≤82	61.8 (49.8–71.7)			
>82	70.7 (59.8–79.2)			
Time to MR4, mo		<0.001		
≤12 (ref.)	78.6 (69.0–85.5)		1.611 (0.856–3.032)	0.140
>12	46.4 (32.0–59.6)			
Duration of MR4, mo		0.081		
≤61	57.0 (44.8–67.4)			
>61	75.3 (64.9–83.1)			
Low-dose of TKI		0.320		
Yes	55.8 (30.3–75.2)			
No	66.7 (58.0–74.0)			

2-G TKI, second-generation tyrosine kinase inhibitor.

#### 3.4.1 Outcomes after molecular relapse

In the present study, 58 patients lost MMR at a median time of 4 months (range, 1–23 months), with 42 (72.4%) patients losing MMR within 6 months, 54 (93.1%) patients lost MMR within 12 months. At the last follow-up, 56/57 patients who were restarted TKI regained MMR with a median time of 3 months (range, 1–29), and 47 patients regained MR4 with a median time of 4 months (range, 1–11). For the full-dose group, 49 patients were retreated with TKI (imatinib 400 mg/d, *n* = 27; imatinib 600 mg/d, *n* = 2; imatinib 200 mg/d, *n* = 4; dasatinib 100 mg, *n* = 2; nilotinib 300 mg/d, *n* = 2; nilotinib 600 mg/d, *n* = 9; Flumatinib 600 mg/d, *n* = 3), of which 49 patients achieved MMR and 41 patients achieved MR4. For the low-dose group, eight patients were restarted on imatinib treatment (200 mg/d, *n* = 4; 300 mg/d, *n* = 2, 400 mg/d, *n* = 2) and six of them obtained MR4. At last follow-up, one patient died of non-CML-related disease, one patient was not in MMR, 18 patients were in MMR, and 170 patients were in MR4 or deeper response.

#### 3.4.2 TKI withdrawal syndrome

In this study, 14 patients (7.4%) developed transient musculoskeletal pain within 12 months after TKI discontinuation, of which 10 patients were on imatinib and 4 patients were on nilotinib. 13 patients were in standard-dose group, only one patient was in the de-escalation group. Thirteen patients reported a grade 1–2 TKI withdrawal syndrome and one patient reported a grade 3 TKI withdrawal syndrome. Three patients lost MMR after the occurrence of musculoskeletal pain.

## 4 Discussion

Currently, long-term TKI treatment is no longer indicated for all patients with CML, as some patients who obtain DMR can safely discontinue TKI. For example, the DASFREE study showed the 1-year TFR rate was 48%, which was 58% in the ENESTop study (Mahon et al., 2018; Shah et al., 2020). The EURO-SKI study with the largest number of patients reported a TFR rate of 61% at 6 months, 50% at 2 years, and 49% at 3 years (Saussele et al., 2018). In addition, several results from real-world studies demonstrated the feasibility of TKI discontinuation. An observational study reported an estimated TFR at 12 months of 69% for Italian patients (Fava et al., 2019). A study from a Spanish research group described a TFR rate at 4 years of 64% (Hernandez-Boluda et al., 2018). Recently, a Swedish discontinuation study showed that 62.2% of patients maintained TFR at the last follow-up (Flygt et al., 2021). However, limited data are available on TKI discontinuation for Chinese CML patients. In the present study, a total of 190 patients who discontinued TKI medication were included in this study. The estimated TFR was 68.8%, and 65.5% at 12 and 24 months, comparing favorably to the TFR rate of approximately 50% in most clinical studies (Saussele et al., 2018). The rate is also similar to the TFR rate reported in Italian, Spanish, and Swedish patients (Hernandez-Boluda et al., 2018; Fava et al., 2019; Flygt et al., 2021). After discontinuation, most relapse occurred within the first 6 months. In this study, 72.4% of patients lost MMR within 6 months, and 93.1% of patients lost MMR within 12 months. One patient lost MMR at 23 months after TKI discontinuation. A recent study showed an estimated rate of molecular recurrence after 2 years of discontinuing imatinib of 18% (Rousselot et al., 2020). Hence, prolonged molecular monitoring is still necessary for patients who have not relapsed in the early phase of drug discontinuation. Most patients (56/57) who were restarted TKI regained MMR, and 47 patients regained MR4 with a median time of 4 months (range, 1–11). The results within the Chinese population showed that TKI discontinuation is safe and feasible also outside controlled clinical trials.

At present, dose optimization is increasingly emphasized as an important part of individualized treatment for CML patients with different demands. In the present study, 27 patients experienced dose reduction before stopping TKI for adverse events (*n* = 17), desire to stop TKI (*n* = 8), and finical burden (*n* = 2). Patients with low-dose TKI treatment had a TFR of 63.8% at 6 months and 55.8% at 12 months. Similarly, patients with full-dose displayed a TFR of 78.4% and 70.3% at 6 months and 12 months, respectively. In the study of Cayssials et al. (2020), the TFR rate at 12 months was 56.8% for full-dose and 80.8% for the low-dose group. However, the DESTINY study reported patients experiencing dose reduction had a TFR rate of 72% after 24 months off TKI (Clark et al., 2019), which appeared to be preferable to the TFR rate of 50% in the EURO-SKI study (Saussele et al., 2018). Claudiani et al. (2021) reported a TFR of 74.1% among patients who have experienced dose reduction. In Claudiani et al. (2021) study, the median duration of MR4 before stopping TKI was 6.1 years, significantly longer than that in the EURO-SKI of 3.1 years. Thus, they suggested that the increased TFR in the low-dose group might be related to the prolonged duration of MR4 to stopping TKI. Recently, Iurlo et al. (2022) investigated the effect of TKI reduction on TFR in a large multicenter cohort of 194 patients with low-dose TKI. Iurlo et al. (2022) study, 71.1% patients were still in TFR after a median follow-up of 29.2 months, which is better than the 62% reported in a recent Italian study with 293 patients who discontinued TKI after a median follow-up of 34 months (Fava et al., 2019; Iurlo et al., 2022). Still, it remains controversial whether the dose reduction before TKI withdrawal can lead to an improvement in TFR. 81.5% (22/27) low-dose patients experienced half of the standard-dose treatment. Moreover, we found the degree of TKI dose reduction was not related to the successful discontinuation, in agreement with the study of Cayssials et al. (2020). Fassoni et al. (2018) developed a mathematical model based on selected patients in the IRIS and the CML-IV study, suggesting that a TKI dose reduction of at least 50% does not affect long-term treatment. In addition, we found that the duration of dose reduction also did not affect the acquisition of TFR. Interestingly, Iurlo et al. (2022) found TFR was significantly better after dose reduction due to adverse events than those with dose de-escalation after DMR achievement. Consistent with the literature, our data showed that dose reductions do not hinder the achievement of TFR, and TFR might be independent of the degree of reduction of TKI.

In the univariate analysis, the type of TKI, TKI resistance, line of therapy, and time to MR4, was the clinical variable significantly associated with molecular recurrence. Multivariate analysis showed patients with TKI resistance presented with significantly lower TFR rates than those without resistance. Similarly, imatinib resistance is an important risk factor for molecular relapse in the DADI trial (Okada et al., 2018). In ENESTnd 10-year analysis, patients treated with first-line nilotinib achieved a higher rate of TFR eligibility compared to first-line imatinib (Kantarjian et al., 2021). A previous study also showed that first-line 2-3G TKIs compared to imatinib were significantly associated with a better TFR (Etienne et al., 2020). In the present study, we compared the TFR of patients treated with imatinib continuously and patients transferred to 2G-TKI from imatinib. We found patients transferred to 2G-TKI treatment for imatinib resistance or non-resistance reasons had remarkably higher TFR rates than those who did not. In addition, patients who switched to 2G-TKI therapy for imatinib resistance or non-resistance reasons achieved similar TFR rates compared to patients treated with 2G-TKI in the first line. Likewise, patients treated with nilotinib as first-line and second-line experienced a similar TFR rate (58% vs. 51.6%) at 48 weeks in the ENESTfreedom study and the ENESTop study (Mahon et al., 2018; Radich et al., 2021). A recent study showed that patients treated with 2G-TKI as first-line treatment and at discontinuation had a lower net probability of TKI re-initiation (*p* = 0·017) compared with patients treated with imatinib (Flygt et al., 2021). In general, our results combined with data from other studies suggested that for patients with a need for discontinuation, second-generation TKI therapy may yield higher TFR rates than imatinib, especially for imatinib-resistant patients.

Several studies reported some patients developed or worsened musculoskeletal pain after discontinuing TKI, suggesting the presence of withdrawal syndrome. For example, the KID study reported that 30% of patients underwent withdrawal syndrome after TKI withdrawal (Lee et al., 2016). Berger et al. (2019) analyzed the withdrawal syndrome after stopping TKIs by combining the patients included in the STIM2 and EURO-SKI trials and showed that 23.2% of patients developed withdrawal syndrome. In addition, 21% of patients in the DESTINY study reported new musculoskeletal symptoms during the dose-reduction phase (Clark et al., 2017). In the present study, 14 patients (7.4%) developed transient musculoskeletal pain within 12 months after TKI discontinuation. Thirteen patients reported a grade 1–2 TKI withdrawal syndrome and one patient reported a grade 3 TKI withdrawal syndrome. Three patients lost MMR after the occurrence of musculoskeletal pain.

## 5 Conclusion

In conclusion, this retrospective study showed TKI discontinuation in the real-world is as feasible and safe as in clinical studies. Indeed, dose optimization is an important consideration in individualized treatment for patients with different demands. Moreover, we found TFR rates were not impaired in patients experiencing dose reduction before TKI discontinuation compared to patients with full-dose TKI. Our data may provide a basis for the safety and feasibility of dose optimization before drug withdrawal. For patients with a need for discontinuation, second-generation TKI therapy may yield higher TFR rates than imatinib, especially for imatinib-resistant patients. However, our study still has limitations as a retrospective study. In addition, only 27 patients discontinued low-dose TKI in this study, and the data on discontinuation after dose reduction may be biased. Some patients with a long history of disease did not have BCR-ABL1 mutations test, so we did not analyze the effect of BCR-ABL1 mutations on TFR. As real-world data are still increasing, more studies are needed to accumulate data in daily practice to confirm the feasibility of dose reduction.

## Data Availability

The original contributions presented in the study are included in the article/Supplementary Material, further inquiries can be directed to the corresponding authors.
